# A new species of *Amphitecna* (Bignoniaceae) endemic to Chiapas, Mexico

**DOI:** 10.3897/phytokeys.65.8454

**Published:** 2016-06-15

**Authors:** Andres Ernesto Ortiz-Rodriguez, Carlos Manuel Burelo Ramos, Héctor Gomez-Dominguez

**Affiliations:** 1 Instituto de Ecología, A.C., Departamento de Biología Evolutiva, Xalapa, Veracruz, México; 2 Herbario UJAT, División Académica de Ciencias Biológicas, Universidad Juárez, Autónoma de Tabasco, Villahermosa Tabasco, México; 3 Herbario Eizi Matuda (HEM) Facultad de Ciencias Biológicas, Universidad de Ciencias y Artes de Chiapas, Tuxtla Gutiérrez, Chiapas, México

**Keywords:** Crescentieae, karst forest, zona sujeta a protección ecológica “La Pera”

## Abstract

*Amphitecna loreae* Ortiz-Rodr. & Burelo, **sp. nov.** (Bignoniaceae), a new species endemic to the karst rainforest in southern Mexico, is described and illustrated. The new species differs from the other species of *Amphitecna* by the combination of cauliflorous inflorescences, larger flowers, buds rounded at apex, and globose-ellipsoid rather than acuminate fruits. A key to the Mexican species of *Amphitecna* is presented.

## Introduction

Bignoniaceae (calabash tree family) includes about 82 genera and approximately 900 species of trees, shrubs and woody vines distributed mainly in tropical areas around the world (Lohmann and Ulloa 2006). The most recent tribal classification of Bignoniaceae (Fischer et al. 2004), recognizes seven tribes: Bignonieae, Coleeae, Crescentieae, Eccremocarpeae, Oroxyleae, Tecomeae, and Tourrettieae. However, phylogenetic analysis based on molecular characters (Olmstead et al. 2009) shows that many of the above tribes, as traditionally had been recognized, do not represent monophyletic groups. Based on this phylogenetic hypothesis (Olmstead et al. 2009), the 82 genera of Bignoniaceae can be organized in the tribes Bignonieae, Catalpeae, Jacarandeae, Oroxyleae, Tecomeae, and Tourrettieae. In addition, a strongly supported clade informally named Crescentiina is recognized (Olmstead et al. 2009, Collevatti and Dornelas 2016).

The Crescentiina clade contains approximately 34 genera and 300 species, and it is formed by two subclades corresponding to the *Tabebuia* alliance and the Paleotropical clade (a group of genera traditionally assigned to Tecomeae and Coleeae) (Olmstead et al. 2009). The *Tabebuia* alliance, is a lineage endemic to the Neotropics and consists of 14 genera and 147 species of trees and shrubs, from which stands a small clade of three genera traditionally assigned to the tribe Crescentieae, *Amphitecna*, *Crecentia*, and *Parmentiera*, which together comprise nearly 36 species of trees distributed in Central America, northern Colombia and the Greater Antilles (Gentry 1980, Grose and Olmstead 2007a).

The genus *Amphitecna* is easily differentiated from *Crecentia* and *Parmentiera* by the combination of simple, alternate leaves and the greenish flowers with the lobes of the petals fused (Gentry 1980). The genus comprises about 20 species (Grose and Olmstead 2007b), most of them known to be restricted to a few localities. In Mexico, the genus *Amphitecna* is particularly diverse and consists of roughly 10 species, all of them having their southernmost distribution in Guatemala and Belize.

During the course of several botanical explorations in southern Mexico, a species of *Amphitecna* with a unique combination of features differing from all other members of the genus was collected in a karst forest of Chiapas. In this paper, this interesting species is described and illustrated and its affinities with other species of *Amphitecna* are discussed. Furthermore a key to Mexican species of *Amphitecna* is presented.

## Materials and methods

In order to confirm the status of this new species we visited and reviewed the specimens of *Amphitecna* deposited in herbaria XAL, HEM and CHIP (Thiers 2016). Also, we consulted the digitized type specimens available at JSTOR Global Plants (http://plants.jstor.org/). The putative new species was recognized using the unique combination of features criteria (Donoghue 1985) through comparisons with morphologically similar species and literature review (Gentry 1980). Finally, description of the species was elaborated following terminology presented in Hickey (1973).

We assessed the conservation status by calculating the extent of occurrence (EOO) and the area of occupancy (AOO) using the GeoCAT tool (Bachman et al. 2011) and applying the IUCN Red List Categories and criteria (IUCN 2001).

Additionally, coordinates of occurrence data were assembled for the new species herein described and for the morphologically similar species, which were obtained from the Global Biodiversity Information Facility (GBIF; http://www.gbif.org/species/4003073), supplemented with records from field collection and with information available in the herbarium specimens. Then climate layers were obtained at a 30 arc-sec (c. 1 km2) resolution from the WorldClim database (Hijmans et al. 2005) and for all occurrence records, we extracted data from 19 climatic variables using ArcView v3.2 (ESRI, Redlands, CA, USA). Using these data, we performed a principal components analysis (PCA) using a correlation matrix with PAST ver. 3.06 (Hammer et al. 2001) to explore patterns of climatic differentiation between species.

## Taxonomic treatment

### Amphitecna loreae

Taxon classificationPlantaeLamialesBignoniaceae

Ortiz-Rodr. & Burelo sp. nov.

urn:lsid:ipni.org:names:77155494-1

Figures 1 , 3

#### Type.

Mexico. Chiapas, Municipio de Berriozábal, zona sujeta a protección ecológica “La Pera”, predio “Peña Flor” camino Berriozábal- Vista Hermosa-El Cairo, km. 12 desvío al Pozo Turipache, 1068 m, 16°51'50.6"N, 93°19'51.7"W, 05 March 2012 (fl, fr) *Ortiz-Rodríguez A. E 0178* (holotype HEM; isotypes: UJAT, XAL).

#### Diagnosis.

**Figure 1.**
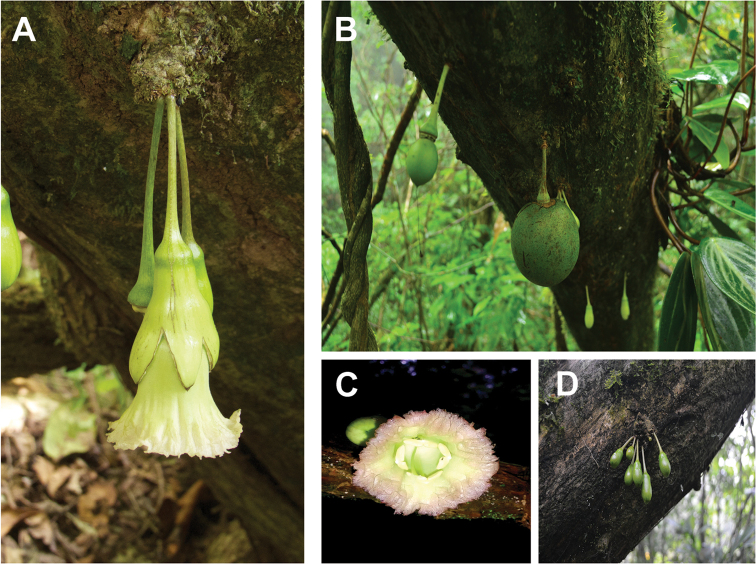
*Amphitecna loreae* sp. nov. **A** cauliflorous flowers with trilabiate calyx. **B** broadly elliptical to spherical fruits **C** corolla **D** buds rounded at apex. Photographs by Andres E. Ortiz-Rodriguez (**A** and **C**) and Marcos Escobar (**B** and **D**).

*Amphitecna loreae* is distinguishable from the other species of *Amphitecna* by a combination of its cauliflorous inflorescences, large flowers, buds rounded at apex, and broadly elliptical to spherical rather than acuminate fruits. *Amphitecna tuxtlensis*, *Amphitecna montana* and *Amphitecna latifolia*, also distributed in Mexico, have afﬁnities with *Amphitecna loreae* and share the cauliflorous inflorescences and leaves less than 50 cm long. However, *Amphitecna tuxtlensis* differs in having the flower buds pointed at the apex and fruits elliptic, acute to acuminate at apex, and *Amphitecna montana* differs in having larger leaves, long pedicellate flowers and elliptical fruits shortly pointed at the tip, while *Amphitecna latifolia* differs in having obovate to widely elliptic leaves, rounded to mucronate at apex with poorly defined petioles (Figure 1).

#### Description.

Trees, 15–25 m and 15–50 cm DBH, the secondary branches terete. *Leaves*, alternate-verticillate, clustered near the apex of the branches, olive-green when dry, glabrous, 10–20 cm long, 2–5 cm wide, oblanceolate to narrowly elliptic, acuminate, subcoriaceous, acute to attenuate basis, midrib slightly raised on the upper surface, prominent on the lower surface; secondary veins 11–14 on a side, slightly raised above, prominent below; petiole short, to 2 cm long, merging with attenuate leaf base. *Inflorescences*, groups of two or three flowers, with an unpleasant odor, which are borne on leafless portions of old branches and along the main trunk (cauliflory). Flower buds, rounded at apex. *Flowers* pendant, pedicel 35–60 mm long; calyx campanulate, 28–38 mm long, more or less coriaceous, evenly 2 to 3-labiate to below the middle, circumscissile; corolla radially symmetric, pale green, tubular-infundibuliform, 48–60 mm long, 30–40 mm wide at the mouth of the tube, the basal part of the corolla a straight tube, 15–25 mm long, the lobes fused in to frilly-margined rim; stamens included, inserted 18–28 mm from base of the tube, the anther thecae divergent, 4–7 mm long, the filaments 18–30 mm long; the staminode, when present, less than 20 mm long, inserted 10–20 mm from base of the tube, sometimes well developed (with one or two small thecae) to give the impression of being a fifth stamen; ovary, up to 3 mm long and 2.5 mm wide, broadly elliptical, glandular-papillose; pistil 40–60 mm long with the stigma bifurcate; disc annular-pulvinate, about 6 mm in diameter; flowers are often found with signs of herbivory, in which the ovule and disc are not present. *Fruits* broadly elliptical to spherical, 70–100 mm long, 60–80 mm wide.

#### Habitat and ecology.

The species is only known from Chiapas, Mexico. It is a rare species at the type locality in the ecological reserve La Pera. The species inhabits the karst areas, mainly in the tropical rainforest. It is a canopy tree and coexists with species of *Quercus lancifolia* Schltdl. & Cham., *Quercus corrugata* Hook., *Calatola costaricensis* Standl., *Spathacanthus hahnianus* Baill, and *Quararibea funebris* (La Llave) Vischer.

#### Phenology.

Mature flowers and fruits were collected in March and April; buds, ripe and immature fruits were observed in the same months.

#### Etymology.

The speciﬁc epithet honors Francisco Lorea Hernández, in recognition of his many important contributions to our knowledge of the Mexican flora.

#### Conservation status.

Currently we lack the necessary information to objectively define the conservation status of *Amphitecna loreae*. However, according to the criteria established by the IUCN, it is possible to tentatively determine that the species is Critically Endangered [CR B1ab (iii)]. Although the only known population of the species is located within a protected natural area, *Amphitecna loreae* appears to be rare ecologically and only eight individuals in one hectare of sampling were recorded (Escobar-Castellanos 2016). The Area of occupancy (AOO) is 12,000 km² and the Extent of occurrence (EOO) is 0.763 km², suggesting a very restricted overall distribution. Furthermore, the tropical rain forest in this region of Chiapas is seriously fragmented and only small remnants persist.

#### Additional specimens examined.

Mexico. Chiapas, Berriozabal: Rancho “El Retiro”, atrás de el rancho “El Zapote”. 13 km al N de Berriozábal camino a Joaquín Miguel Gutiérrez, 16°52'09.2"N, 93°19'26.4"W, 1114 m., 04 May 2014, *M. A. Escobar Castellanos 586* (HEM); same locality, *M. A. Escobar Castellanos 675* (HEM); zona sujeta a protección ecológica “La Pera”, predio “Peña Flor” camino Berriozábal- Vista Hermosa-El Cairo, km. 12 desvío al Pozo Turipache, 16°51'50.6"N, 93°19'51.7"W,1100 m, 16 May 2015, *Y. Licona-Vera 190* (XAL).

#### Discussion.

*Amphitecna loreae* sp. nov. has a combination of characters that clearly separate it from other species of *Amphitecna* : its strictly cauliflorous inflorescences distinguish it from those species with terminal inflorescences (*Amphitecna apiculata* A.H. Gentry, *Amphitecna breedlovei* A.H. Gentry, *Amphitecna donnell-smithii* (Sprague) L.O. Williams, *Amphitecna isthmica* (A.H. Gentry) A.H. Gentry, *Amphitecna molinae* L.O. Williams and *Amphitecna steyermarkii* (A.H. Gentry) A.H. Gentry).

**Figure 2.**
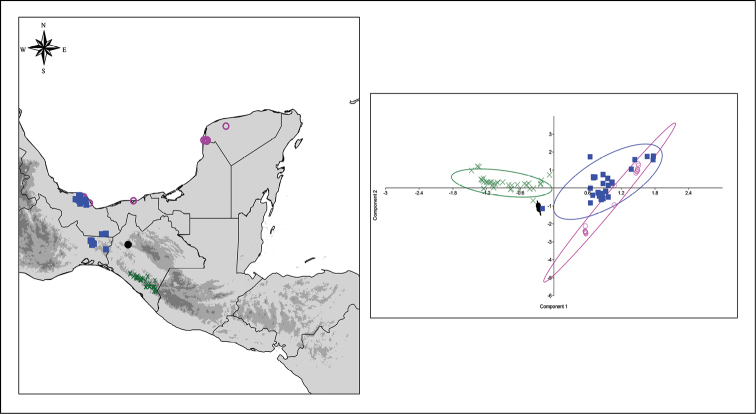
Distribution range and climatic preferences of *Amphitecna loreae* and related species. *Amphitecna latifolia* (purple circles) *Amphitecna montana* (green cross), *Amphitecna loreae* (black dots) and *Amphitecna tuxtlensis* (blue squares). In colours similar to those of the species the 95% confidence ellipses produced by PCA analysis.

The four cauliferous species discussed in the diagnoses have different distribution ranges with different climatic preferences (Figure 2). *Amphitecna tuxtlensis* has two disjunct populations in Veracruz, one in the area of the Los Tuxtlas and another in the Uxpanapa-Chimalpas region, where it inhabits the tropical rainforest. *Amphitecna montana* is distributed along the Sierra Madre de Chiapas and inhabits the cloud forest above 1200 m. *Amphitecna latifolia* is distributed intermittently in areas near to the Atlantic coast of Mexico, where it inhabits mainly in riparian vegetation and mangrove associations. In contrast, *Amphitecna loreae* is endemic to Chiapas and it is known only from a single locality at the municipality of Berriozabal, Chiapas. The species grows on a karstic zone at approximately 900–1,150 m and it inhabits the tropical rainforest (Table 1).

**Figure 3.**
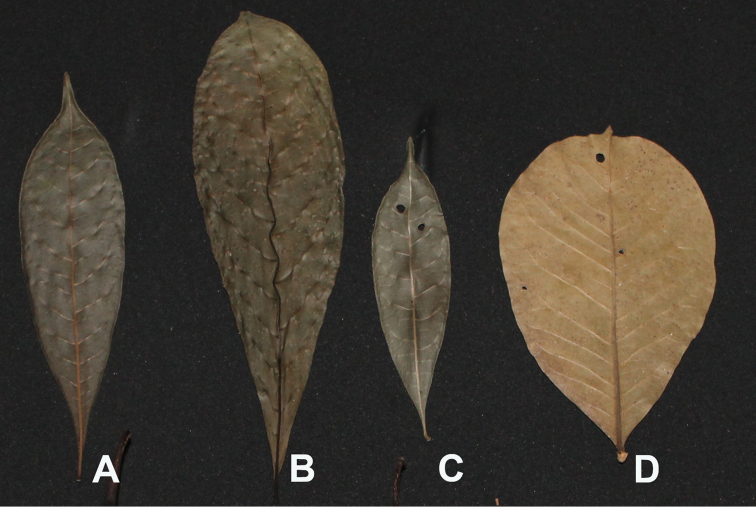
Leaf variation in *Amphitecna loreae* and related species. **A** *Amphitecna tuxtlensis* (*H. Gomez 3710* HEM) **B** *Amphitecna montana* (*N. Martinez 927* HEM) **C** *Amphitecna loreae* (*M. Escobar 586* HEM) and **D** *Amphitecna latifolia* (*E. Ucan E 251* XAL).

**Table 1.** Comparison of diagnostic morphological characters of *Amphitecna loreae* with its close relatives.

Characters	*Amphitecna latifolia*	*Amphitecna montana*	*Amphitecna tuxtlensis*	*Amphitecna loreae*
**Habit**	Tree to 10 m tall	Large tree, 10-20 m tall	Tree, 5-15 m tall	Large tree, 10-25 m tall
**Leaf length**	to 19 cm	to 34 cm	to 18 cm	to 20 cm
**Leaf width**	to 11 cm	to 11 cm	to 5 cm	to 5 cm
**Petiole**	poorly defined	clearly differentiated	defined	defined
**Leaf shape**	Broadly obovate	Oblanceolate to narrowly obovate	Oblanceolate	Oblanceolate
**Leaf apex**	rounded to acute, usually apiculate	acute to short-acuminate	acuminate	acuminate
**Length of the flower pedicel**	to 36 mm	to 100 mm	to 26 mm	to 60 mm
**Tip of flower buds**	rounded	rounded	Pointed	rounded
**Fruit shape**	broadly elliptical to spherical	Oblong-ovoid or ellipsoid	ellipsoid	broadly elliptical to spherical
**Fruit apex**	rounded (rare shortly pointed)	shortly pointed to acute	acute to acuminate	rounded
**Habitat**	always near sea level, mostly in mangrove associations and flooded vegetation	Mountain cloud forest	Tropical rain forest	Tropical rain forest
**Distribution**	Mexico (Campeche, Tabasco, Veracruz and Yucatan); Central America, West Indies to Venezuela and Ecuador	Mexico (Chiapas); Guatemala	Mexico (Veracruz and Oaxaca)	Mexico (Chiapas)

### Key to the Mexican species of *Amphitecna* (modified from Gentry 1980)

**Table:** 

1	Terminal inflorescences	**2**
–	Cauliflorous inflorescences (borne on leafless portions of old branches and along the main trunk)	**5**
2	Calyx spathaceous with a sharp acumen	***Amphitecna steyermarkii***
–	Calyx bilabiate or trilabiate	**3**
3	Corolla tubular less than 1 cm wide at the mouth of tube	***Amphitecna apiculata***
–	Corolla campanulate more than 1 cm wide at the mouth of tube	**4**
4	Leaves membranaceous; corolla less than 3 cm long	***Amphitecna donnell-smithii***
–	Leaves chartaceous to coriaceous; corolla more than 3 cm long	***Amphitecna breedlovei***
5	Leaves mostly 50–100 cm long, clustered near tip of twigs; small trees, 2–7 m, simple or few branched stem	**6**
–	Leaves less than 40 cm long, alternate; medium and large sized trees,10–25 m, branched	**7**
6	Corolla less than 2 cm wide at the mouth of tube; pedicels to 4 cm long	***Amphitecna macrophylla***
–	Corolla more than 2 cm wide at the mouth of tube; pedicels to 1 cm long	***Amphitecna regalis***
7	Fruits ovoid to narrowly oblong-ellipsoid, apiculate at apex	**8**
–	Fruits ellipsoid to spherical, rounded at apex or very inconspicuously apiculate	**9**
8	Secondary venation impressed below leaves and conspicuously whitish-margined; petiole poorly demarcated, to 1 cm long; flower buds rounded to shortly pointed	***Amphitecna silvicola***
–	Secondary venation prominent below leaves; and not whitish-margined; petiole 1–2 cm long; flower buds pointed	***Amphitecna tuxtlensis***
9	Trees to 10 m tall; leaves obovate to wide elliptic, rounded to mucronate at apex with poorly defined petioles; restricted to coastal ecosystems	***Amphitecna latifolia***
–	Large trees, to 25 m tall; leaves oblanceolate to narrowly obovate, acute to acuminate at apex with defined petioles; tropical rain forest or cloud forest	**10**
10	Leaves, 34 ×11 cm; petiole to 4 cm long; pedicels to 10 cm long; forests above 1200 m	***Amphitecna montana***
–	Leaves, 20 ×5 cm; petiole short, less than 2 cm; pedicels to 6 cm long; forests below 1000 m	***Amphitecna loreae***

## Supplementary Material

XML Treatment for Amphitecna loreae
